# Supplementary material for: Development and validation of the leisure lifestyle and satisfaction assessment: A comprehensive tool for evaluating leisure engagement
Source: PLoS One. 2025 Dec 31;20(12):e0338099. doi: 10.1371/journal.pone.0338099 (PMC12755763; doi:10.1371/journal.pone.0338099)
Supplement: S1 File — (DOCX) [file pone.0338099.s001.docx]

Leisure lifestyle section.

Please answer based on your actual participation in leisure activities over the past month.

1. Please indicate how important engaging in leisure activities is to you.

**extremely unimportant**

**extremely important**

**1**

**2**

**3**

**4**

**5**

1. Please write down the leisure activities you currently engage in or schedule, along with their frequency and time slots.

Example: swimming , 2 times per week/month (time slots: morning/afternoon/evening)

1. , times per week/month (time slots: morning/afternoon/evening)
2. , times per week/month (time slots: morning/afternoon/evening)
3. , times per week/month (time slots: morning/afternoon/evening)
4. Please circle the leisure activities you are interested in pursuing.

**Audiovisual activities:** watching movies, watching television, watching videos (e.g., on YouTube), surfing social networks (e.g., Facebook), playing video games, listening to music, and karaoke.

**Learning activities:** reading, writing, language learning, photography, performing arts, tea ceremonies, book clubs, and attending lectures.

**Outdoor activities:** walking, traveling, riding a bicycle, using roller skates, bird-watching, and camping.

**Intellectual pursuits:** chess, playing cards, mahjong, sudoku, puzzles, magic, brain teasers, and Rubik’s Cube.

**Artistic interests:** playing musical instruments, art and cultural exhibitions, handicrafts, dancing, painting, and appreciating drama.

**Sports activities:** ball sports, hiking, swimming, jogging, fitness, aerobic exercise, qigong, tai chi, Yuanji dance, and Neidan exercise.

**Hobbies:** gardening, pet keeping, collecting items, and stamp collecting.

**Social activities:** gathering with friends, family gatherings, hot spring bathing, shopping, religious activities, and volunteer work.

Leisure satisfaction section

1. My ability to organize and plan leisure activities.
2. The variety of leisure activities I can choose from.
3. The variety of leisure activity equipment or facilities I can choose from.
4. The frequency of my participation in leisure activities.
5. I have sufficient time to participate in leisure activities each time.
6. My performance in leisure activities.
7. My physical strength and endurance during leisure activities.
8. The enjoyment I derive from engaging in leisure activities.
9. The sense of achievement I gain from participating in leisure activities.
10. My expenditure on leisure activities.
11. The safety of the venue where I engage in leisure activities.
12. The convenience of transportation to the venue where I engage in leisure activities.
13. My interpersonal performance during leisure activities.
14. Overall satisfaction with my participation in leisure activities.
