# Supplementary material for: Development and validation of the leisure lifestyle and satisfaction assessment: A comprehensive tool for evaluating leisure engagement
Source: PLoS One. 2025 Dec 31;20(12):e0338099. doi: 10.1371/journal.pone.0338099 (PMC12755763; doi:10.1371/journal.pone.0338099)
Supplement: Attachment — Submitted filename: Review Report.pdf [file pone.0338099.s004.pdf]

Review Report

I would like to thank the authors for providing a comprehensive response to the study and for making the necessary revisions in line with the reviewers' comments and suggestions. The additional sections have contributed to strengthening the overall integrity of the research.

I believe that supporting this valuable study, which is intended to be introduced to the literature, with a stronger theoretical foundation will enhance its academic rigor. The main focus of the study, which examines individuals with schizophrenia within the context of therapeutic recreation as a functional aspect of leisure, is of significant importance. Therefore, it is recommended that, in the introduction, the concept of leisure be discussed not in its general sense but specifically within the framework of therapeutic recreation. Such an approach would improve both the coherence of the work and its original contribution. The literature review presented in the Procedures section may serve as an important reference point for the authors in this regard.

The main shortcoming of the study appears to stem from the excessive generalization of the topic. For this reason, it is suggested that the structure of the study be reorganized within a stronger theoretical framework. The recommended revisions are not related to analyses or procedures but rather to the need for a more robust theoretical grounding and for addressing leisure from a functional perspective.

When evaluated in terms of limitations, I believe that by removing the fourth limitation, the scope of the study could be expanded. This would also provide a valuable guide for future research.

Overall, the study has the potential to make a meaningful contribution to the literature. However, the suggested revisions would substantially strengthen its academic depth and enhance its value to the field.
