# Supplementary material for: Development and validation of the leisure lifestyle and satisfaction assessment: A comprehensive tool for evaluating leisure engagement
Source: PLoS One. 2025 Dec 31;20(12):e0338099. doi: 10.1371/journal.pone.0338099 (PMC12755763; doi:10.1371/journal.pone.0338099)
Supplement: Attachment — Submitted filename: Response to Reviewers 8-7-2025.docx [file pone.0338099.s003.docx]

Reviewer #1: I hope this letter finds you well. I had the opportunity to review your article titled, “Development and validation of the leisure lifestyle and satisfaction assessment: A comprehensive tool for evaluating leisure engagement”, which was submitted Plos One.

1. Abstract

- The abstract is structurally relatively consistent and includes elements of the research purpose, methods, results, and conclusion, so the flow of the writing is good.

**Reply:**

**Thank you for your valuable feedback.**

- In particular, the research purpose (development of a new assessment tool, LISA) and the main assessment methods (including Rasch analysis) were clearly described.

**Reply:**

**Thank you for your valuable feedback.**

- The development of a leisure participation measurement tool for patients with schizophrenia is a topic with practical and clinical usefulness and is considered to have high academic/clinical contribution.

**Reply:**

**Thank you for your valuable feedback.**

- However, some sentences after the middle are redundant or have ambiguous word choices, making the meaning unclear.

**Reply:**

**We have deleted the redundant words and revised the sentences in the abstract section as follows: “*Subsequently, 200 people with schizophrenia from one psychiatric center completed the two sections of the measure.*”** (lines 29-31, page 2, abstract section)

- The fact that the study subjects were limited to patients from a single institution and the limitations in generalizability were not mentioned at all in the abstract.

**Reply:**

**We have added this limitation in the abstract section as follows: “*****The participants of this study were recruited from a single institution and we excluded people with schizophrenia with severe cognitive impairments, which may restrict generalizability. Future research is warranted to recruit people with schizophrenia from multiple institutions to cross-validate our findings.*”** (lines 40-43, page 3, abstract section)

2. Introduction

- The introduction clearly states the purpose of the study by emphasizing the importance of leisure activities in improving the quality of life of patients with schizophrenia.

**Reply:**

**Thank you for your valuable feedback.**

- It is judged that the relevant literature persuasively expresses that leisure has a positive effect on cognitive, emotional, and social functions.

**Reply:**

**Thank you for your valuable feedback.**

- It is considered to be academically outstanding that the concepts of leisure lifestyle and leisure satisfaction were independently defined and the influence of these two factors on mental health and quality of life was explained.

**Reply:**

**Thank you for your valuable feedback.**

- It is considered desirable to suggest a starting point for research by mentioning existing tools such as COPM and LSS.

**Reply:**

**Thank you for your valuable feedback.**

- At the end of the introduction, the research objective of ‘developing the LISA tool to evaluate the leisure lifestyle and satisfaction of patients with schizophrenia’ is clearly stated, which is expected to help readers understand.

**Reply:**

**Thank you for your valuable feedback.**

- However, although sufficient evidence has been presented on the effectiveness of leisure activities, it is judged necessary to explain the specific limitations of existing leisure measurement tools (e.g. COPM, LSS, etc.).

**Reply:**

**The COPM and LSS are used to assess leisure satisfaction. However, these two measures do not provide detailed information related to leisure lifestyle****, such as regular participation in leisure activities, frequency of performing leisure activities, and leisure activities that individuals would like to engage in. We have revised related information in the introduction section as follows: “*****While the COPM and LSS assess leisure satisfaction, these two measures do not provide detailed information related to leisure lifestyle, such as regular participation in leisure activities, frequency of performing leisure activities, and leisure activities that individuals would like to engage in.*”** (lines 75-78, page 5, introduction section)

- Lack of critical context as to why new tools are needed.

**Reply:**

**Clinicians and researchers must gather comprehensive information on both leisure lifestyle and leisure satisfaction when designing interventions. However, previous measures have not simultaneously captured these two critical sections (i.e., leisure lifestyle and leisure satisfaction) in a holistic manner. Therefore, the purpose of this study was to develop a new measure, the leisure LIfestyle and SAtisfaction measure (LISA), which can help understand leisure lifestyle and leisure satisfaction in people with schizophrenia simultaneously.**

**We have revised related information in the introduction section as follow: “*Clinicians and researchers must gather comprehensive information on both leisure lifestyle and leisure satisfaction when designing interventions*** ***in clinical and research settings.*** ***However, current measures have not simultaneously captured these two critical sections (i.e., leisure lifestyle and leisure satisfaction) in a holistic manner, which results in an incomplete understanding of the full spectrum of leisure engagement. Therefore, the purpose of this study was to develop a new measure, the leisure LIfestyle and SAtisfaction measure (LISA), which can help understand leisure lifestyle and leisure satisfaction in people with schizophrenia simultaneously, ultimately contributing to their overall well-being and quality of life.*”** (lines 78-86, page 5-6, introduction section)

- We hope to emphasize a critical awareness of what specific academic or clinical gaps in the existing literature this study seeks to fill.

**Reply:**

**Measures like the COPM and LSS only assess leisure satisfaction, which could not simultaneously capture information related to leisure lifestyle, such as regular participation in leisure activities, frequency of performing leisure activities, and leisure activities that individuals would like to engage in. This gap underscores the need for a more comprehensive measure, which can assess both leisure lifestyle and life satisfaction in a holistic manner. Clinicians and researchers must gather comprehensive information on both leisure lifestyle and leisure satisfaction when designing interventions in clinical and research settings.**

**We have revised related information in the introduction section as follows: “*****While the COPM and LSS assess leisure satisfaction, these two measures do not provide detailed information related to leisure lifestyle, such as regular participation in leisure activities, frequency of performing leisure activities, and leisure activities that individuals would like to engage in. Clinicians and researchers must gather comprehensive information on both leisure lifestyle and leisure satisfaction when designing interventions in clinical and research settings. However, current measures have not simultaneously captured these two critical sections (i.e., leisure lifestyle and leisure satisfaction) in a holistic manner, which results in an incomplete understanding of the full spectrum of leisure engagement.*”** (lines 75-83, page 5, introduction section)

- We need to move beyond the simple ‘need’ level and make a compelling case for ‘why existing methodologies are not sufficient.’

**Reply:**

**While the COPM and LSS assess leisure satisfaction, they do not provide a comprehensive picture of leisure lifestyle, such as regular participation in leisure activities, frequency of performing leisure activities, and leisure activities that individuals would like to engage in. This lack of detail hampers the ability of clinicians and researchers to fully understand and address the diverse needs of people with schizophrenia. The existing measures are insufficient for capturing the full spectrum of leisure engagement.**

**We have revised related information in the introduction section as follows: “*While the COPM and LSS assess leisure satisfaction, these two measures do not provide detailed information related to leisure lifestyle, such as regular participation in leisure activities, frequency of performing leisure activities, and leisure activities that individuals would like to engage in. Clinicians and researchers must gather comprehensive information on both leisure lifestyle and leisure satisfaction when designing interventions in clinical and research settings. However, current measures have not simultaneously captured these two critical sections (i.e., leisure lifestyle and leisure satisfaction) in a holistic manner, which results in an incomplete understanding of the full spectrum of leisure engagement.*”** (lines 75-83, page 5, introduction section)

3. Method

- The effort to secure the content validity and empirical legitimacy of the tool through a three-stage systematic tool development process (literature review → expert consultation → cognitive interview → main survey → validity/reliability verification) is considered appropriate and methodologically sound.

**Reply:**

**Thank you for your valuable feedback.**

- The use of scale analysis based on the Rasch model has strengths in psychometric approaches, and the attempt to explain the internal consistency of the instrument through reliability and unidimensionality verification is impressive.

**Reply:**

**Thank you for your valuable feedback.**

- The criteria for selecting participants (DSM-5-based diagnosis, MMSE ≥ 22, stable condition, etc.) are clear, and it is considered highly desirable to limit the subjects to those with secured cognitive ability.

**Reply:**

**Thank you for your valuable feedback.**

- It is judged that the fact that the study was conducted separately as a cognitive interview (n=15) and a main survey (n=200) increased the design precision.

**Reply:**

**Thank you for your valuable feedback.**

- Three leisure lifestyle items and 15 leisure satisfaction items are described separately, and the detailed item composition is also sufficiently explained.

**Reply:**

**Thank you for your valuable feedback.**

- The results of statistical verification are presented in detail, including confirmation of unidimensionality through Rasch analysis (PCA eigenvalue = 2.2), appropriate infit/outfit range (0.75~1.27), reliability coefficient (Rasch reliability = 0.90), and absence of ceiling/floor effect, sufficiently supporting reliability and validity.

**Reply:**

**Thank you for your valuable feedback.**

- However, the type of research design was not clearly specified.

**Reply:**

**This study employed a methodological, cross-sectional design focusing on the development and validation of the LISA. We have added related information in the methods section as follows: “*****This study employed a methodological, cross-sectional design focusing on the development and validation of the LISA.*”** (lines 90-91, page 6, methods section)

- This study is classified as a measurement tool development study, but the absence of an explicit statement of the type of design may limit the reader's understanding.

**Reply:**

**We have added the type of design in the methods section as follows: “*This study employed a methodological, cross-sectional design focusing on the development and validation of the LISA.*”** (lines 90-91, page 6, methods section)

- Since statistical basis for sample size (e.g. minimum required number of cases for Rasch analysis) is not provided, it is difficult for readers to independently judge the validity of the test.

**Reply:**

**A sample size of 200 was recommended when calibrating item parameters under the Rasch model^1,2^. We have added related information in the methods section as follows: “*****A sample size of 200 was recommended when calibrating item parameters under the Rasch model [25, 26].*”** (lines 97-98, page 6, methods section)

- The theoretical basis or prior research on the criteria for classifying leisure activities is unclear.

**Reply:**

**The eight categories were derived by synthesizing the classification reported in previous leisure studies^3,4^, which groups activities based on their underlying purpose, such as entertainment, learning, physical health, creativity, intellectual stimulation, personal interest, and social interaction. These frameworks provide a theoretical basis for distinguishing leisure activities according to intention or motivation associated with participation.**

**We have added related information in the methods section as follows: “*Leisure activities cover a wide range, and thus we classified the leisure activities of the third item*** ***in the leisure lifestyle section into eight categories.*** ***The eight categories were derived by synthesizing the classification reported in previous leisure studies [27, 28], which groups activities based on their underlying purpose, such as entertainment, learning, physical health, creativity, intellectual stimulation, personal interest, and social interaction. These frameworks provide a theoretical basis for distinguishing leisure activities according to intention or motivation associated with participation.*** ***The eight categories were as follows: (1) audiovisual activities: watching movies, watching television, watching videos (e.g., on YouTube), surfing social networks (e.g., Facebook), playing video games, listening to music, and karaoke; (2) learning activities: reading, writing, language learning, photography, performing arts, tea ceremonies, book clubs, and attending lectures; (3) outdoor activities: walking, traveling, riding a bicycle, using roller skates, bird-watching, and camping, (4) intellectual pursuits: chess, playing cards, mahjong, sudoku, puzzles, magic, brain teasers, and Rubik’s Cube; (5) artistic interests: playing musical instruments, art and cultural exhibitions, handicrafts, dancing, painting, and appreciating drama; (6) sports activities: ball sports, hiking, swimming, jogging, fitness, aerobic exercise, qigong, tai chi, Yuanji dance, and Neidan exercise; (7) hobbies: gardening, pet keeping, collecting items, and stamp collecting; (8) social activities: gathering with friends, family gatherings, hot spring bathing, shopping, religious activities and volunteer work (Appendix A).*”** (lines 118-137, page 7-8, methods section)

- The leisure lifestyle (3 items) section relies only on technical summaries without statistical analysis, raising questions about the structural integrity of the entire tool.

**Reply:**

**The three items of the leisure lifestyle section were intentionally designed as descriptive indicators rather than as a unidimensional latent construct subjected to psychometric analysis. We have added related information in the methods section as follows: “*****After expert consultation, the leisure lifestyle section included three items: (1) level of importance of participating in leisure activities (5-point scale: 1 = extremely unimportant to 5 = extremely important); (2) regular participation in leisure activities and frequency of engagement; and (3) leisure activities respondents would like to engage in.*** ***The three leisure lifestyle items were designed as independent indicators to describe patterns of leisure engagement rather than to represent a single latent construct subjected to psychometric analysis. Thus, descriptive statistics were used to summarize responses.*”** (lines 112-118, page 7, methods section)

4. Results

- Table 1 clearly presents basic demographic characteristics such as gender, age, education level, medication type, cognitive function (MMSE), and disease severity (CGI-S), thereby explaining how to judge the representativeness and heterogeneity of the sample.

**Reply:**

**Thank you for your valuable feedback.**

- Based on the numerical values such as the average MMSE (26.6 points) and the average CGI-S (3.4 points), it can be seen that the overall cognitive level and symptom severity level of the subjects were good, so it was determined that the tool's self-reporting potential was secured.

**Reply:**

**Thank you for your valuable feedback.**

- It is very impressive that the respondents emphasized practical significance by evaluating the importance of leisure activities (average 4.0), presenting the top 10 activities they actually performed, and the top 10 activities they hoped to participate in.

**Reply:**

**Thank you for your valuable feedback.**

- The lifestyle items are judged to provide vivid data on user experience by combining qualitative and quantitative data.

**Reply:**

**Thank you for your valuable feedback.**

- The results of Rasch analysis are statistically well presented.

**Reply:**

**Thank you for your valuable feedback.**

- However, since only the frequency is presented for this item without statistical analysis, it is judged impossible to evaluate its structural validity as a measurement indicator.

**Reply:**

**The leisure lifestyle items were developed as independent indicators to capture the descriptive aspects of leisure engagement. As they were not intended to form a single latent dimension, no structural validity analysis was conducted for these items. We have added related information in the methods section as follows: “*The three leisure lifestyle items were designed as independent indicators to describe patterns of leisure engagement rather than to represent a single latent construct. Thus, descriptive statistics were used to summarize responses.*”** (lines 115-118, page 7, methods section)

- There is a lack of quantitative discussion on the interpretation of the results. For example, the meaning or clinical applicability of the Rasch reliability value of 0.90 is not specifically interpreted.

**Reply:**

**We have added the meaning and clinical applicability of the Rasch reliability value of 0.90 in the discussion section as follows: “*Rasch reliability denotes the extent to which a measure is*** ***free from measurement error, reflecting the precision of the measurement [36].*** ***A Rasch reliability value of 0.90 indicates excellent reliability, meaning that the leisure satisfaction section can reliably differentiate people with schizophrenia with different levels of leisure satisfaction. For clinical applications, a high reliability (≥ 0.90)*** *s****upports the confident use of the scores for making individual-level decisions.*”** (lines 223-227, page 13, discussion section)

5. Discussion

- The discussion clarifies the purpose of the study by relatively clearly defining the connection between the research purpose (development of a leisure lifestyle and satisfaction measurement tool for patients with schizophrenia) and the main results (ensuring reliability and unidimensionality based on Rasch analysis, no ceiling/floor effect).

**Reply:**

**Thank you for your valuable feedback.**

- It is judged that the strength of the logical development is that it emphasizes the two-sided evaluation function (interest activities vs. satisfaction) of the LISA tool and naturally highlights the possibility of actual clinical use.

**Reply:**

**Thank you for your valuable feedback.**

- Some of the results, such as the proportion of audiovisual activities and the desire for social activities, implicitly suggest that they are similar to existing studies, thereby showing a certain degree of literary connection and thus making an academic contribution.

**Reply:**

**Thank you for your valuable feedback.**

- The part in the latter half of the discussion that emphasized that LISA is simple and easy to use in clinical settings, taking only 10 to 15 minutes, can be said to be a good example of the possibility of practical use.

**Reply:**

**We have added that the LISA should be considered as a practical and useful tool in the discussion section as follows:** **“*The LISA is a self-reported measure used to comprehensively assess leisure in people with schizophrenia. It has three features that are applicable to people with schizophrenia…******Third, it is simple and easy to use in clinical setting, as the items are straightforward and completion time is short (10-15 minutes for the leisure lifestyle section and 5-10 minutes for the leisure satisfaction section). Therefore, the LISA can be considered as a practical and useful tool, as it enables efficient data collection without placing an excessive burden on the examinees and examiners.*”** (lines 233-234 and 239-243, page 13-14, discussion section)

- Along with its advantages as a self-reporting tool, its ability to collect information on leisure activities of patients with schizophrenia in a structured form is considered a strength as a clinical tool.

**Reply:**

**We have added the strength of the ability to collect information on leisure activities in a structured form in the discussion section as follows:** **“*The LISA is a self-reported measure used to comprehensively assess leisure in people with schizophrenia. It has three features that are applicable to people with schizophrenia…******Second, it collects information on leisure activities in a structured form, which allows clinicians and researchers to obtain organized and clinically relevant data to guide intervention planning.*”** (lines 233-234 and 237-239, page 13-14, discussion section)

- We clearly recognize the limitations of the sample size, which is limited to participants from a single institution, and the exclusion of patients with severe cognitive impairment. However, we highly value the suggestion of future multi-institutional studies and the possibility of introducing proxy reports.

**Reply:**

**Thank you for your thoughtful and encouraging comment.**

- It is considered a desirable discussion to emphasize that while existing tools focused on leisure satisfaction, this tool has a practical contribution by measuring leisure participation values and preferences as well.

**Reply:**

**Indeed, it is important to emphasize that while existing tools focus on leisure satisfaction, the LISA provides a practical contribution by measuring leisure participation values and preferences. We have added related information in the discussion section as follows: “*The LISA is a self-reported measure used to comprehensively assess leisure in people with schizophrenia. It has three features that are applicable to people with schizophrenia. First, the LISA simultaneously assesses leisure interests and satisfaction.*** ***While existing measures emphasize leisure satisfaction, the LISA additionally assesses leisure participation values and preferences.*”** (lines 233-237, page 13, discussion section)

- It is judged that there is a lack of consideration of the cross-cultural applicability of LISA and its expandability, such as its applicability to various mental illness groups.

**Reply:**

**This study did not examine the cross-cultural applicability of the LISA and its applicability to various mental illness groups. We have added this limitation in the discussion section as follows: “*This study has four notable limitations…******Third, this study did not examine the applicability of the LISA across different cultures and mental illness populations. Future research should explore the applicability of the LISA in diverse cultural contexts and investigate its psychometric properties in other mental illness populations.*”** (lines 244 and 252-255, page 14, discussion section)

- It is judged that there is a lack of explanation as to what theoretical models (e.g., behavioral theory, self-determination theory, quality of life model, etc.) these contributions are based on and extended.

**Reply:**

**Although the LISA was not developed based on a single formal theoretical model, it was developed based on existing empirical classifications and conceptual works in leisure research^3,4^. The leisure activities of the LISA were categorized according to purposes of leisure participation identified in previous empirical studies, such as entertainment, learning, physical health, creativity, intellectual stimulation, personal interest, and social interaction. This approach reflects a synthesis of established classifications in leisure research and provides a conceptually grounded framework for understanding the purposes and preferences underlying leisure engagement.**

**We have added this limitation in the discussion section as follows: “*This study has four notable limitations…******Fourth, the development of the LISA was not based on a specific theoretical model. Although the categorization of leisure activities was based on previous empirical classifications, the absence of a formal guiding framework may limit the theoretical coherence of the tool.*”** (lines 244 and 256-258, page 14-15, discussion section)

6. Conclusion

- It clearly summarizes the achievement of the core objective of this study (development of a LISA tool that can simultaneously evaluate the leisure lifestyle and satisfaction of patients with schizophrenia).

**Reply:**

**Thank you for your valuable feedback.**

- In particular, it was mentioned that ‘construct validity, Rasch reliability, and no ceiling/floor effects’ were secured for the leisure satisfaction section, and the measurement completeness of the tool was concisely emphasized, which reaffirmed the necessity of this study.

**Reply:**

**Thank you for your valuable feedback.**

- It is positive that the clinical practicality and interventional potential were briefly mentioned through the fact that the research results can be utilized by ‘clinicians and researchers to tailor leisure interventions.’

**Reply:**

**Thank you for your valuable feedback.**

- However, the conclusion does not include any mention of the limitations of the study (single-center recruitment, exclusion of subjects with cognitive impairment, etc.), which may give the impression of over-exaggerating the scope and generalizability of the study.

**Reply:**

**We have added the limitations of the study in the conclusions section as follows: “*The LISA is a new measure designed to assess leisure lifestyle and satisfaction.*** ***The leisure lifestyle section provides descriptive information on individuals’ perceived importance of leisure, frequency of participation, and preferred leisure activities, offering clinically relevant insights to guide intervention planning. The leisure satisfaction section has satisfactory construct validity, Rasch reliability, and no ceiling/floor effects. The LISA can provide valuable information for clinicians and researchers to tailor leisure interventions for people with schizophrenia.*** ***This study was conducted at a single institution and excluded people with schizophrenia with severe cognitive impairments, which may restrict the generalizability of our results. Future research is needed to validate the LISA across diverse clinical settings and populations to strengthen its applicability.*”** (lines 261-270, page 15, conclusions section)

- It is judged that there is a lack of a clear vision for the expandability and development direction of the research, as there are no suggestions for follow-up research (multi-institutional research, application to various groups, modification and supplementation of tools, etc.).

**Reply:**

**We have added information on future research in the conclusions section as follows: “*This study was conducted at a single institution and excluded people with schizophrenia with severe cognitive impairments, which may restrict the generalizability of our results. Future research is needed to validate the LISA across diverse clinical settings and populations to strengthen its applicability.*”** (lines 267-270, page 15, conclusions section)

Reviewer #2: The topic addressed in the study is undoubtedly of interest and potential value to the field. However, the manuscript appears to lack sufficient methodological rigor, particularly in terms of statistical analysis. The procedures related to the development and validation of the measurement tool are either insufficiently described or altogether absent, which undermines the study’s credibility and replicability. In addition, the introduction section requires further elaboration. A more comprehensive and well-structured overview of the research background is necessary to clearly situate the study within the existing body of literature. In particular, the justification for the development of such a measurement instrument should be articulated in greater detail, emphasizing its theoretical and practical significance for the field.

**Reply:**

**For the methodology, we conducted expert review, expert consultation, and cognitive interviews for the two sections of the LISA (leisure lifestyle and leisure satisfaction). The leisure satisfaction section was examined for construct validity, Rasch reliability, and ceiling/floor effects. The items of the leisure lifestyle section was designed to describe patterns of leisure engagement rather than to represent a single latent construct subjected to psychometric analysis. Thus, descriptive statistics were used to summarize responses. We have added related information in the methods section as follows: “*The LISA was designed as a self-reported measure in three stages. The first stage involved a literature review that focused on leisure measures for people with disabilities. We designed the LISA with two sections: leisure lifestyle and leisure satisfaction. The leisure lifestyle section aimed to explore the personal value of engaging in leisure activities and preferences for leisure activities in both the present and future. The leisure satisfaction section aimed to assess the level of satisfaction derived from participating in the leisure activities. The second stage involved expert consultation. Eight experts, including occupational therapists, psychiatrists, and researchers specializing in psychometric properties provided comments on the suitability, wording, and relevance of the content, as well as scoring.*** ***After expert consultation, the leisure lifestyle section included three items: (1) level of importance of participating in leisure activities (5-point scale: 1 = extremely unimportant to 5 = extremely important); (2) regular participation in leisure activities and frequency of engagement; and (3) leisure activities respondents would like to engage in. The three leisure lifestyle items were designed as independent indicators to describe patterns of leisure engagement rather than to represent a single latent construct subjected to psychometric analysis. Thus, descriptive statistics were used to summarize responses...*** ***Cognitive interviews were conducted with 15 people with schizophrenia who met the inclusion and exclusion criteria. Cognitive interviews were conducted to gather feedback from people with schizophrenia on the clarity and comprehensibility of the content regarding leisure lifestyle and leisure satisfaction items. The third stage was the validation of the leisure satisfaction section of the LISA. Construct validity (e.g., unidimensionality), Rasch reliability, and ceiling/floor effects were evaluated.*”** (lines 103-118 and 140-145, page 6-8, methods section)

**In response to your suggestion that a more comprehensive and well-structured overview of the research background is necessary to clearly situate the study within the existing body of literature, we have revised the Introduction to better contextualize the need for the LISA. Regarding the research background for conducting this study, existing leisure measures for people with schizophrenia, such as the Canadian Occupational Performance Measure (COPM) and the Leisure Satisfaction Scale (LSS), primarily focus on leisure satisfaction but do not capture essential elements of leisure lifestyle, including regular participation, frequency of engagement, and desired activities. Recognizing that both leisure lifestyle and satisfaction are critical for meaningful engagement and intervention planning, this study aimed to develop the Leisure Lifestyle and Satisfaction Measure (LISA), which simultaneously addresses both dimensions. We have added related in the introduction section as follows: “*While the COPM and LSS assess leisure satisfaction, these two measures do not provide detailed information related to leisure lifestyle, such as regular participation in leisure activities, frequency of performing leisure activities, and leisure activities that individuals would like to engage in. Clinicians and researchers must gather comprehensive information on both leisure lifestyle and leisure satisfaction when designing interventions in clinical and research settings. However, current measures have not simultaneously captured these two critical sections (i.e., leisure lifestyle and leisure satisfaction) in a holistic manner, which results in an incomplete understanding of the full spectrum of leisure engagement. Therefore, the purpose of this study was to develop a new measure, the leisure LIfestyle and SAtisfaction measure (LISA), which can help understand leisure lifestyle and leisure satisfaction in people with schizophrenia simultaneously, ultimately contributing to their overall well-being and quality of life.*”** (lines 75-86, page 5-6, introduction section)

**From a practical perspective, the LISA provides clinicians with structured, time-efficient information that can directly be informative for intervention planning. We have added related in formation in the discussion section as follows: “*The LISA is a self-reported measure used to comprehensively assess leisure in people with schizophrenia. It has three features that are applicable to people with schizophrenia…Second, it collects information on leisure activities in a structured form, which allows clinicians and researchers to obtain organized and clinically relevant data to guide intervention planning. Third, it is simple and easy to use in clinical setting, as the items are straightforward and completion time is short (10-15 minutes for the leisure lifestyle section and 5-10 minutes for the leisure satisfaction section). Therefore, the LISA can be considered as a practical and useful tool, as it enables efficient data collection without placing an excessive burden on the examinees and examiners.*”** (lines 233-234 and 237-243, page 13-14, discussion section)

**Although the LISA was not developed based on a single formal theoretical model, it was developed based on existing empirical classifications and conceptual works in leisure research^3,4^. The leisure activities of the LISA were categorized according to purposes of leisure participation identified in previous empirical studies, such as entertainment, learning, physical health, creativity, intellectual stimulation, personal interest, and social interaction. This approach reflects a synthesis of established classifications in leisure research and provides a conceptually grounded framework for understanding the purposes and preferences underlying leisure engagement. To acknowledge this limitation, we have added relation information in the discussion section as follows: “*This study has four notable limitations…Fourth, the development of the LISA was not based on a specific theoretical model. Although the categorization of leisure activities was based on previous empirical classifications, the absence of a formal guiding framework may limit the theoretical coherence of the tool.*”** (lines 244 and 256-258, page 14-15, discussion section)

References

1.	He W. CAT field-test item calibration sample size: How large is large under the Rasch model? Global Journal of Human-Social Science. 2015;151:73-79.

2.	Chiu EC, Lee SC, Kuo CJ, Lung FW, Hsueh IP, Hsieh CL. Development of a Performance-Based Measure of Executive Functions in Patients with Schizophrenia. PLoS One. 2015;10:e0142790.

3.	Scott D, Willits FK. Adolescent and adult leisure patterns: a reassessment. J Leis Res. 1998;30:319-330.

4.	Lloyd KM, Auld CJ. The role of leisure in determining quality of life: issues of content and measurement. Soc Indic Res. 2002;57:43-71.
