# Supplementary material for: Development and validation of the leisure lifestyle and satisfaction assessment: A comprehensive tool for evaluating leisure engagement
Source: PLoS One. 2025 Dec 31;20(12):e0338099. doi: 10.1371/journal.pone.0338099 (PMC12755763; doi:10.1371/journal.pone.0338099)
Supplement: Attachment — Submitted filename: Response to Reviewers 10-22-2025.docx [file pone.0338099.s005.docx]

Reviewer #2: Review Report

I would like to thank the authors for providing a comprehensive response to the study and for making the necessary revisions in line with the reviewers’ comments and suggestions. The additional sections have contributed to strengthening the overall integrity of the research.

I believe that supporting this valuable study, which is intended to be introduced to the literature, with a stronger theoretical foundation will enhance its academic rigor. The main focus of the study, which examines individuals with schizophrenia within the context of therapeutic recreation as a functional aspect of leisure, is of significant importance. Therefore, it is recommended that, in the introduction, the concept of leisure be discussed not in its general sense but specifically within the framework of therapeutic recreation. Such an approach would improve both the coherence of the work and its original contribution. The literature review presented in the Procedures section may serve as an important reference point for the authors in this regard.

**Reply:**

**We appreciate your recommendation** **to frame the concept of leisure more specifically within the context of therapeutic recreation, rather than in a general sense. We have added related information in the introduction section as follows: “*****Leisure is not only an activity, but also a structured and purposeful process that contributes to individuals’ functional independence and quality of life within the framework of therapeutic recreation [8, 12].*** ***This perspective highlights the clinical significance of assessing leisure and aligns with the goals of therapeutic interventions for people with schizophrenia.*”** (lines 64-68, pages 4-5, introduction section)

The main shortcoming of the study appears to stem from the excessive generalization of the topic. For this reason, it is suggested that the structure of the study be reorganized within a stronger theoretical framework. The recommended revisions are not related to analyses or procedures but rather to the need for a more robust theoretical grounding and for addressing leisure from a functional perspective.

**Reply:**

**Thank you for this insightful comment. While the LISA was not developed based on a single formal theoretical model, its development was supported by existing literature emphasizing the purposeful and health-promoting aspects of leisure participation. We have revised related information in the discussion section as follows: “*The LISA is a self-reported measure used to comprehensively assess leisure in people with schizophrenia. It has three features that are applicable to people with schizophrenia. First, the LISA simultaneously assesses leisure interests and satisfaction. While existing measures emphasize leisure satisfaction, the LISA additionally assesses leisure participation values and preferences.*** ***The development of the LISA was supported by existing literature emphasizing the purposeful and health-promoting aspects of leisure.*”** (lines 237-242, pages 13-14, discussion section)

When evaluated in terms of limitations, I believe that by removing the fourth limitation, the scope of the study could be expanded. This would also provide a valuable guide for future research.

**Reply:**

**We have removed the fourth limitation.**

Overall, the study has the potential to make a meaningful contribution to the literature. However, the suggested revisions would substantially strengthen its academic depth and enhance its value to the field.

**Reply:**

**We sincerely appreciate the reviewer’s thoughtful and encouraging comments regarding the potential contribution of our study.**
